# Posterior fossa extra-axial variations of medulloblastoma: a pictorial review as a primer for radiologists

**DOI:** 10.1186/s13244-021-00981-z

**Published:** 2021-04-06

**Authors:** Abdulaziz M. Al-Sharydah, Abdulrahman Hamad Al-Abdulwahhab, Sari Saleh Al-Suhibani, Wisam M. Al-Issawi, Faisal Al-Zahrani, Faisal Ahmad Katbi, Moath Abdullah Al-Thuneyyan, Tarek Jallul, Faisal Mishaal Alabbas

**Affiliations:** 1 Diagnostic and Interventional Radiology Department, Imam Abdulrahman Bin Faisal University, King Fahd Hospital of the University, AlKhobar City, Eastern Province Saudi Arabia; 2 Neurosurgery Department, Imam Abdulrahman Bin Faisal University, King Fahd Hospital of the University, AlKhobar City, Eastern Province Saudi Arabia; 3 Radiodiagnostics and Medical Imaging Department, King Fahd Military Medical Complex, Dhahran City, Eastern Province Saudi Arabia; 4 Emergency Department, Imam Abdulrahman Bin Faisal University, King Fahd Hospital of the University, Alkhobar City, Eastern Province Saudi Arabia; 5 Neurosurgery Department, King Fahd Specialist Hospital, Dammam City, Eastern Province Saudi Arabia

**Keywords:** Cerebellopontine angle, Lateral cerebellum, Tentorium cerebelli, Foramen magnum, Medulloblastoma

## Abstract

Manifestations of an atypical variant of medulloblastoma of the posterior fossa in extra-axial locations have been reported, and key questions concerning its interpretation have been raised previously. This review illustrated the clinico-radiological and histopathological features of the posterior fossa extra-axial medulloblastoma and described possible management strategies. We thoroughly reviewed all atypical anatomical locations of medulloblastoma reported within the posterior fossa and extra-axial spaces. The main characteristics of diagnostic imaging and histopathological results, primarily the distinctive radiopathological characteristics, were summarized to distinguish between intra- and extra-axial medulloblastoma, or pathologies mimicking this tumor. Most cases of posterior fossa extra-axial medulloblastoma have been reported in the cerebellopontine angle, followed by the tentorial and lateral cerebellar locations. The dural tail sign, which is commonly observed in meningioma, is rarely seen in intra- or extra-axial medulloblastoma and might be associated with other benign or malignant lesions. In addition to magnetic resonance imaging, the proposed new imaging techniques, including advances in modern neuroimaging modalities, were discussed, as potentially efficient modalities for characterizing extra-axial medulloblastoma. Radionuclide imaging and magnetic resonance perfusion imaging are practical alternatives to limit the number of differential diagnoses. We believe that medulloblastoma cases are likely under-reported because of publication bias and frequent tumors in unusual locations. Addressing these issues would help establish a more accurate understanding of this entity.

## Key points

- Medulloblastoma is a common brain tumor; therefore, understanding its variations is crucial.
- Neuroimaging is helpful in the preoperative neuroaxis evaluation and postoperative assessment of medulloblastoma.
- Identifying and categorizing metastatic disease during diagnosis is paramount for effective therapy.
- Deciphering this challenging diagnosis can reflect positively on a patient’s prognosis.

## Background

Medulloblastomas, which are undifferentiated embryonal neuroepithelial tumors, originate from primitive multipotent cells of the cerebellum and spread to the germ cell migration tract [1]. Other sites of origin of medulloblastomas have also been reported, including the lateral medullary velum and the cerebellar flocculus [2]. The most recent World Health Organization (WHO) Classification from 2016 integrates a modular diagnostic approach with the incorporation of genetically defined entities of medulloblastomas, regardless of their anatomical locations. According to this classification, there are four genetic (molecular) groups of medulloblastoma: the wingless (WNT)-activated and the Sonic Hedgehog (SHH)-activated groups, and the groups numerically designated as “group 3” and “group 4” [3].

Notably, medulloblastomas are common malignancies in pediatric patients, accounting for 25% of all brain tumors of childhood, typically arising from the cerebellar vermis. In contrast, adulthood medulloblastomas are typically observed within the paramedian region or laterally within the cerebellar hemisphere, and account for < 1% of primary brain tumors among adults [1].

Recently, advanced neuroimaging techniques have revealed the presence of medulloblastoma in extra-axial locations [1, 2]. The hypothesis behind the origin of extra-axial variations remains controversial. One study postulated that they originated from primary multipotential cells in the cerebellum and propagated to the germ cell [1]. Other reports have suggested a possible origin from the cerebellar flocculus migration tract or the outer granular layer in the neuroepithelial roof of the fourth ventricle [2, 4]. Clinically, medulloblastomas manifest with nonspecific symptoms, such as headache, fatigue, vomiting, and cerebellar dysfunction. During disease progression, symptoms of increased intracranial pressure (i.e., lethargy, vomiting, seizures, and vision and behavior changes) predominate [5].

Radiologically, medulloblastoma appears as a contrast-enhanced hyperdense region on a computed tomography (CT) scan, often compressing the fourth ventricle; iso- to hypointense on T1-weighted magnetic resonance (MR) images; and hyperintense on T2-weighted MR images [2]. A characteristic diffusion restriction is predominantly observed on MR images because of its high cellularity and a high nuclear-to-cytoplasmic ratio [6]. MR spectroscopy typically reveals a small taurine peak, a high choline peak, an increase in the choline/creatinine and choline/N-acetyl aspartate (NAA) ratios, and a decreased NAA peak [6, 7]. Primitive evidence suggested group-specific spectral patterns, with high taurine peaks and low levels of lipids in “group 3” and “group 4,” and high choline and lipid peaks in SHH-activated medulloblastoma with only trace or absent taurine peaks [8]. Microscopic analyses indicated that adult and pediatric variants of medulloblastoma appear identical, with the desmoplastic variant occurring predominantly in adulthood [9, 10]. Medulloblastomas are characterized by the presence of heterogenous densely packed blue cell tumors, with round to oval, highly hyperchromic nuclei surrounded by scant cytoplasm. In addition, DNA microarrays reveal a distinct gene expression pattern, suggesting that medulloblastoma is not a primitive neuroectodermal tumor [11].

Combined therapy, consisting of a maximally radical surgery followed by chemotherapy and adjuvant irradiation of the entire central nervous system, is typically recommended [12]. Prognosis is essentially dependent on the genetic and molecular subtype, with the worst prognosis observed in “group 3” and the best observed in the WNT-activated subtype [13]. This review elucidated the characteristics of the posterior fossa extra-axial variations of medulloblastoma in comparison with the typical intra-axial medulloblastoma in terms of their clinical features, imaging characteristics, histologic subtypes, and prognosis. Furthermore, we illustrated similar lesions, summarizing the differences based on the characteristics of a variety of neoplastic and non-neoplastic diseases that present as masses in the posterior fossa extra-axial regions.

## Search strategy

We searched for articles published in PubMed until August 2020. Search strings consisted of a combination of the following terms: “medulloblastoma,” “extra-axial,” and “exophytic growth.” The literature search was conducted by three board-certified radiologists among the authors (AMS, AHA, SSS). In total, 27 articles met our eligibility criteria (Table 1). These reports conformed to our definition of extra-axial medulloblastoma, as a process occurring separately and apart from the brain parenchyma, rather than a mere surface-localized exophytic growth. Additionally, we compared these parameters with those of extra-axial mimicking lesions and typical intra-axial medulloblastoma.

**Table 1:** Summary of the posterior fossa extra-axial medulloblastomas documented in the PubMed database, which could aid to distinguish from typical intra-axial medulloblastoma

No	Authors	Origin	Age/sex	CT characteristics	MR characteristics	Management	Follow-up
1	Kumar et al. [2]	CPA	9 years/M	Hyperdense mass on plain scans, with heterogeneous enhancement	T1: hypointense; T2: heterogenous; CEMR: mass enhancement	Tumor excision; chemotherapy and radiotherapy	Died
		CPA	8 years/M	Iso-to hypodense mass with heterogeneous enhancement	–	Tumor excision; radiotherapy	Died
		CPA	20 years/F	–	T1 and T2: heterogeneous signal; CEMR: heterogeneous enhancement	Tumor excision; radiotherapy	Improved
		CPA	24 years/M	Heterogenous mass	–	Subtotal resection; chemotherapy	Died
2	Spina et al. [5]	CPA (2 cases)	22 years/M 26 years/F	–	T2/FLAIR: hyperintense; CEMR: heterogeneous enhancement	Total excision; radiotherapy	Improved
3	Fallah et al. [9]	CPA	47 years/M	Homogenously enhancing mass with well-defined borders	–	Total excision; radiotherapy	–
4	Furtado et al. [10]	CPA	32 years/M	Hyperdense mass on plain scan	T1: hypointense; T2: mixed intensity; CEMR: heterogenous enhancement + dural tail sign; MRS: choline and taurine peak increase and creatine peak decrease	Total excision; radiotherapy	Improved
5	Bhaskar et al. [12]	CPA	Infant/M	Hyperdense mass on plain scans	T1: hypointense; T2: isointense; CEMR: intense homogenous enhancement	Total excision	Died, postoperative day 20
6	Yamada et al. [16]	CPA	19 years/F	Hypoattenuated mass with homogenous enhancement	T1: hypointense; CEMR: mass enhancement	Subtotal resection; immunotherapy and radiotherapy	Improved, with no recurrence
7	Akay et al. [17]	CPA	21 years/M	Heterogenous high attenuation	T1: hypointense; T2: hyperintense; CEMR: heterogeneous	Subtotal resection; chemotherapy and radiotherapy	Improved
8	Jaiswal et al. [18]	CPA (14 cases)	3–53 years/seven M and six F	Heterogeneous attenuation with necrosis	T1: hypointense; T2: hyperintense; CEMR: heterogenous	Seven patients: Total excision; seven patients: subtotal resection; total eight patients received chemotherapy	Follow-up: nine cases Recurrence: two cases Symptom-free: seven cases
9	Becker et al. [22]	CPA (two cases) and tentorial (three cases)	28–52 years/one M and four F	–	Heterogenous signal intensity and enhancement	–	–
10	Meshkini et al. [23]	Lateral cerebellar	19 years/F	–	CEMR: heterogenous intense enhancement with cystic changes	Tumor resection	–
		CPA	7 years/F	–	CEMR: heterogenous intense enhancement with cystic changes	Tumor resection	–
11	Doan et al. [24]	Tentorial	29 years/M	–	CEMR: homogenous enhancement + dural tail sign	Subtotal resection; chemotherapy and radiotherapy	–
12	Presutto et al. [25]	Lateral cerebellar	33 m/M	Mildly hyperattenuating on plain scans, with homogenous enhancement	T2/FLAIR: hyperintense; DWI: restricted diffusion	Total resection	Improved
13	Chung EJ, et al. [26]	Lateral cerebellar	5 years/M	–	T1: isointense; T2: isointense; CEMR: homogeneous enhancement	Tumor excision; radiotherapy	Improved
14	Pant I et al. [45]	CPA	15 years/M		T2: heterogeneous signal intensity with necrotic areas/cystic degeneration; CEMR: heterogeneous enhancement; DWI: restricted diffusion	Tumor resection	–
15	Gil-Salu et al. [49]	CPA	40 years/M	Homogeneously enhancing mass	Homogenous enhancement	Total excision; adjuvant therapy	–
16	Singh et al. [50]	CPA	21 years/M	Heterogeneously non-enhancing mass	Heterogenous signal intensity and enhancement	Total excision	Recurrence and metastasis at 15 months
17	Bahrami et al. [51]	CPA	23 years/M	–	T1: hypointense; T2: hyperintense; CEMR: heterogenous	Total excision; radiotherapy	Improved
18	Mehta et al. [52]	CPA	40 years/M	–	Heterogenous enhancement	Subtotal resection; radiotherapy	Improved
19	Ahn et al. [53]	CPA	9 m/F	–	T1: hypointense; T2: hypointense; CEMR: heterogenous	Subtotal resection; possible chemotherapy and Radiotherapy	Died after 2 months
20	Naim-ur-Rahman et al. [54]	CPA	3 years/F	Heterogeneously enhancing mass	–	Tumor excision	Improved
21	Izycka-Swieszewska et al. [55]	CPA	26 years/F	Homogenous enhancement	T1: hypointense; T2: hyperintense; CEMR: homogenous enhancement	Tumor excision	–
22	Park et al. [56]	CPA	15 years/M	Hyperdense mass causing internal auditory canal dilation	T1: hypointense; T2: hypointense; CEMR: heterogenous	Subtotal resection; chemotherapy and radiotherapy	Improved
23	Santagata et al. [57]	CPA	17 years/F	Hyperdense mass forming a flat surface against the posterior aspect of the left petrous bone and tentorium	CEMR: heterogeneous enhancement	Tumor excision; chemotherapy and radiotherapy	–
24	Nyanaveelan et al. [58]	CPA	5 years/F	Mass eroding the petrous bone	–	Tumor excision; chemotherapy and radiotherapy	–
25	Yoshimura et al. [59]	CPA	29 years/F	–	T1: isointense; T2/FLAIR: Hyperintense; CEMR: No enhancement; DWI: restricted diffusion; MRS: high ratio of choline‐to‐N‐acetyl aspartate	Subtotal resection; chemotherapy and radiotherapy	Improved
26	Cugati et al. [60]	CPA	4 years/F	Contrast-enhancing extra-axial mass in the CPA, centered around the internal acoustic meatus	T1: hypointense; T2: hyperintense; CEMR: intense enhancement	Tumor excision	–
27	Kumar et al. [61]	CPA	9 years/F	Isodense to hypodense mass in the right CPA Homogenous enhancement	T1: hypointense; T2: hyperintense; CEMR: brilliant enhancement	Tumor excision; radiotherapy	Improved

*CEMR* contrast-enhanced magnetic resonance, *FLAIR* fluid-attenuated inversion recovery, *DWI* diffusion-weighted imaging, *MRS* magnetic resonance spectroscopy

## Discussion of clinical review results

**Fig. 1:**
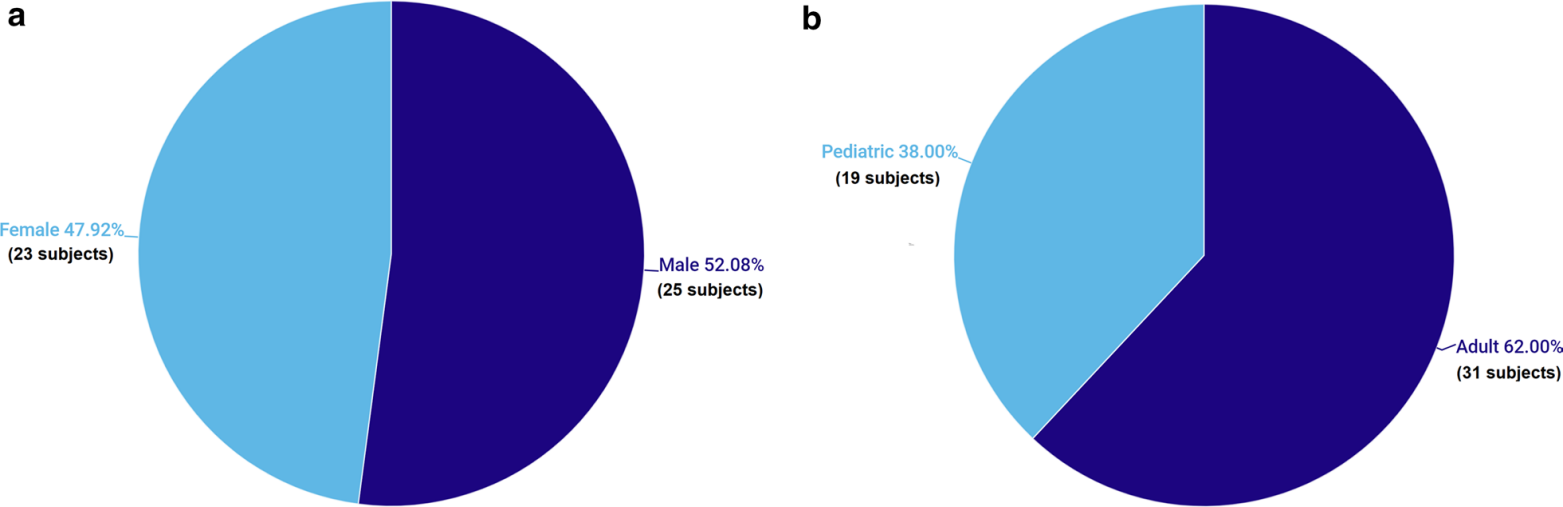
Participant distribution according to sex (**a**) and age (**b**) (pediatric, age < 18 years; adult, age ≥ 18 years)

**Fig. 2:**
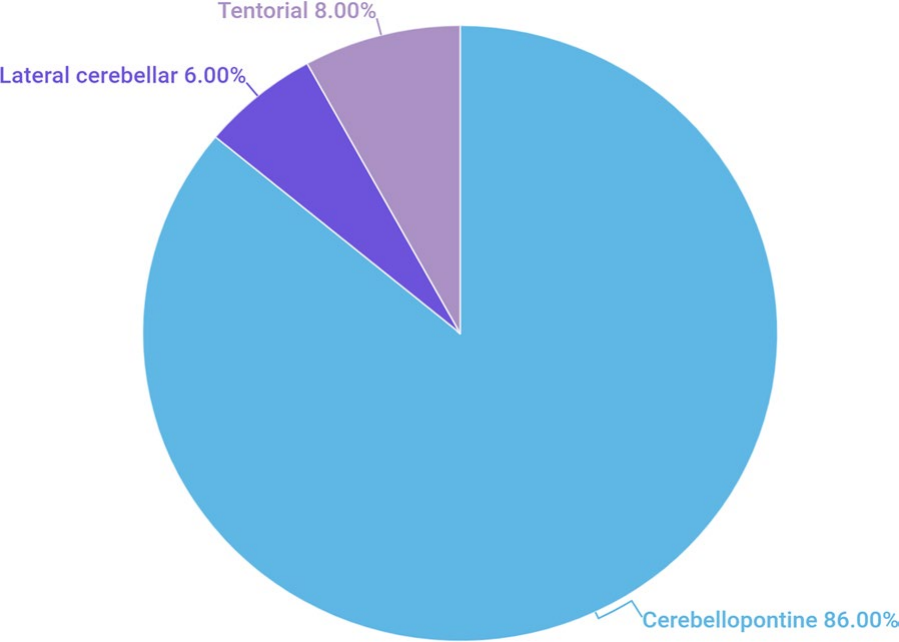
Tumor locations and their corresponding prevalence

**Fig. 3:**
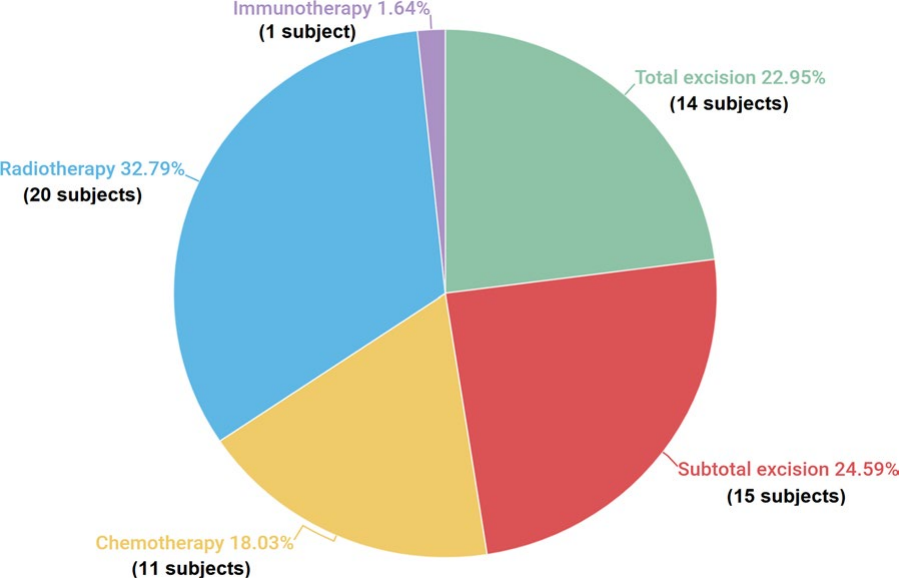
Overview of followed treatment protocols and their corresponding prevalence

“Extra-axial variant of medulloblastoma” is a descriptive term indicating that the lesion originates externally to the brain parenchyma and not from an exophytic outgrowth, beyond the pial surface (i.e., it can originate from the skull, meninges, cranial nerves, and brain appendages) [14]. Based on our extensive literature review, the posterior fossa extra-axial variations of medulloblastoma are classified according to their exact topographic location, with a categorical quantification of the reviewed cases regarding the overall percentage of participants’ sex, age, tumor location, and treatments administered (Figs. 1, 2, 3) for a better comprehension of the disease process, as follows:

### Cerebellopontine angle (CPA) location

The CPA is an extra-axial wedge-shaped cisternal space bounded by the lateral petro-temporal bone, cerebellum, and brain stem medially, as well as by the inferior cranial nerves (CN IX, X, and XI) [15]. Most posterior fossa extra-axial medulloblastomas are reported to be located in the CPA. In total, 43 (86%) of the cases were found from the PubMed database when writing this review. Twenty-six of them were of adult patients (i.e., ≥ 18 years) with a male predominance (25 cases) (Table 1). One hypothesis claimed that these medulloblastomas arise from the remnants of the external granular layer of the cerebellar hemisphere, or from the proliferating residue of the lateral medullary velum, where it meets the CPA [16]. Another hypothesis suggests a lateral spread to the CPA through the foramen of Luschka or from a direct exophytic growth of the lesion in the cerebellum or the pons [16]. No sex predilection has been identified [16].

There are various clinical presentations of CPA medulloblastomas, which are difficult to distinguish from those of other CPA neoplastic or non-neoplastic lesions [16]. Interestingly, the short-term duration of symptoms and progression of brainstem dysfunction and hydrocephalus suggest that the lesions originate from the parenchyma, rather than from extra-axial regions. The commonly encountered clinical manifestations are headache, vomiting, nausea, and cerebellar signs, while hearing deficits and facial nerve involvement are infrequently encountered and may occur as late manifestations [17, 18]. Rarely, extra-axial CPA medulloblastoma may present with a dural-based appearance with hyperostosis, simulating a petrosal meningioma [10]. A few studies have also identified cystic changes, calcifications, and metastases in cases of CPA medulloblastomas [16]. The most common CPA neoplasm is vestibular schwannoma, which accounts for 90% of such cases, followed by meningioma and epidermoid inclusion cyst [17]. The aggressive nature of this tumor and the short duration of its symptoms suggest that a differential diagnosis is needed to distinguish a medulloblastoma and an atypical hypercellular lesion in the CPA (Table 2).

**Table 2:** Clinical and imaging features of common pathologies mimicking medulloblastoma

Diagnosis	Common features
Meningioma	Short duration of symptoms, lack of cranial nerve involvement, and bony hyperostosis A well-demarcated lesion Follow the gray matter signal intensity
Cholesteatoma	A destructive lesion with bony erosion Restricted diffusion on diffusion-weighted images
Nerve sheath tumor	Destruction of the internal auditory canal Follow the signal intensity of the white matter Cystic degeneration and hemorrhagic components are common
Epidermoid inclusion cyst	Follow the signal intensity of CSF with incomplete FLAIR suppression Restricted diffusion and no enhancement on post-contrast images
Metastasis	History of a primary malignant lesion Metastatic work-up to look for other masses
Primary bone tumor	Bony origin with erosion and a calcification or ossification pattern
Choroid plexus papilloma	Well-defined lesion located in the foramen of Luschka Feathery appearance with restricted diffusion and intense postcontrast enhancement
Hemangioblastoma	Young and middle-aged adults High intrinsic vascularity, as evidenced by high rCBV values on perfusion MRI If multiple, strong association with VHL syndrome
Atypical teratoid rhabdoid tumor	Heterogenous solid-cystic mass occurring off-midline in children < 3 years of age
Pilocytic astrocytoma	Cystic lesion with a pathognomonic mural nodule Hypodense on CT images

*CSF* cerebrospinal fluid, *FLAIR* fluid-attenuated inversion recovery, *rCBV* relative cerebral blood volume, *MRI* magnetic resonance images, *VHL* Von Hippel-Lindau, *CT* computed tomography

**Fig. 4:**
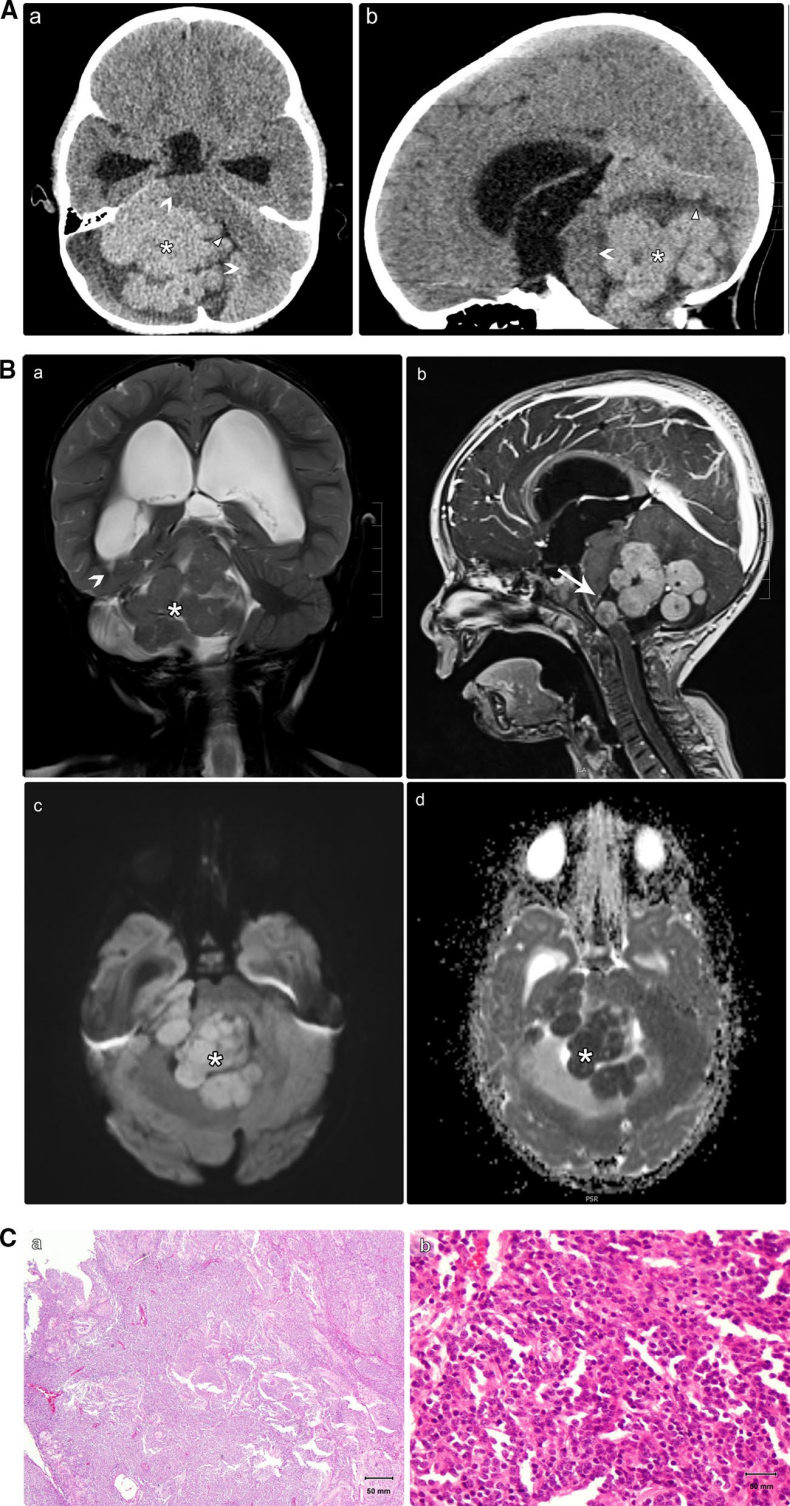
**A** A 7-year-old child was sent to our hospital with a suspected brain tumor. Unenhanced brain CT scan with the axial (a) and sagittal (b) reformats. There is a well-defined hyperdense complex mass in the posterior fossa (asterisks). The mass is centered on the right CPA and surrounded by a fissure of the CSF (cleft sign), proving its extra-axial origin (arrowheads). It induces a mass effect on the ipsilateral cerebellar hemisphere, middle cerebellar peduncle, and pons (benched arrows), resulting in obstructive hydrocephalus. *CPA* cerebellopontine angle, *CSF* cerebrospinal fluid, *CT* computed tomography. **B** Brain MRI images without (a, c-d) and with intravenous contrast (b). There is a sizeable multilobulated mass located at the right CPA (asterisks). The T1-weighted (not shown) and coronal T2-weighted images (a) show iso-signal intensity relative to the gray matter. Following contrast administration, an avid enhancement was depicted (b; sagittal T1 fat saturated), with restricted diffusion on diffusion-weighted images (c, d). This mass exerts a significant mass effect on the brain stem (arrow; b) with the resultant obstructive hydrocephalus (benched arrow; a). The entire neuro-axial imaging was not remarkable (not shown). *MRI* magnetic resonance imaging, *CPA* cerebellopontine angle. **C** Histopathological analysis following surgery with various magnifying powers. (a) A highly cellular tumor was showing undifferentiated cells with variable growth patterns, including small uniform round carrot-shaped cells with hyperchromatic and cytoplasmic nuclei. (b) Distinct fibrillary background composed of cellular processes corresponding to the embryonic class of medulloblastoma

The primary components of CPA medulloblastoma are two molecular subgroups, including WNT-activated and SHH-activated medulloblastoma [19]. CPA medulloblastoma in adult patients appears to have a favorable prognosis. However, a poor prognosis is observed in pediatric patients [20]. We present the case of a 7-year-old child who was diagnosed with the WNT-activated type of medulloblastoma based on imaging and post-surgical pathology results (Fig. 4). Two-year follow-up after adjuvant therapy revealed no recurrence.

### Tentorial location

The cerebellar tentorium is a crescent-shaped dural reflection that extends across the posterior cranial fossa, separating the occipital and temporal brain hemisphere from the cerebellum and the infratentorial brainstem [21].

**Fig. 5:**
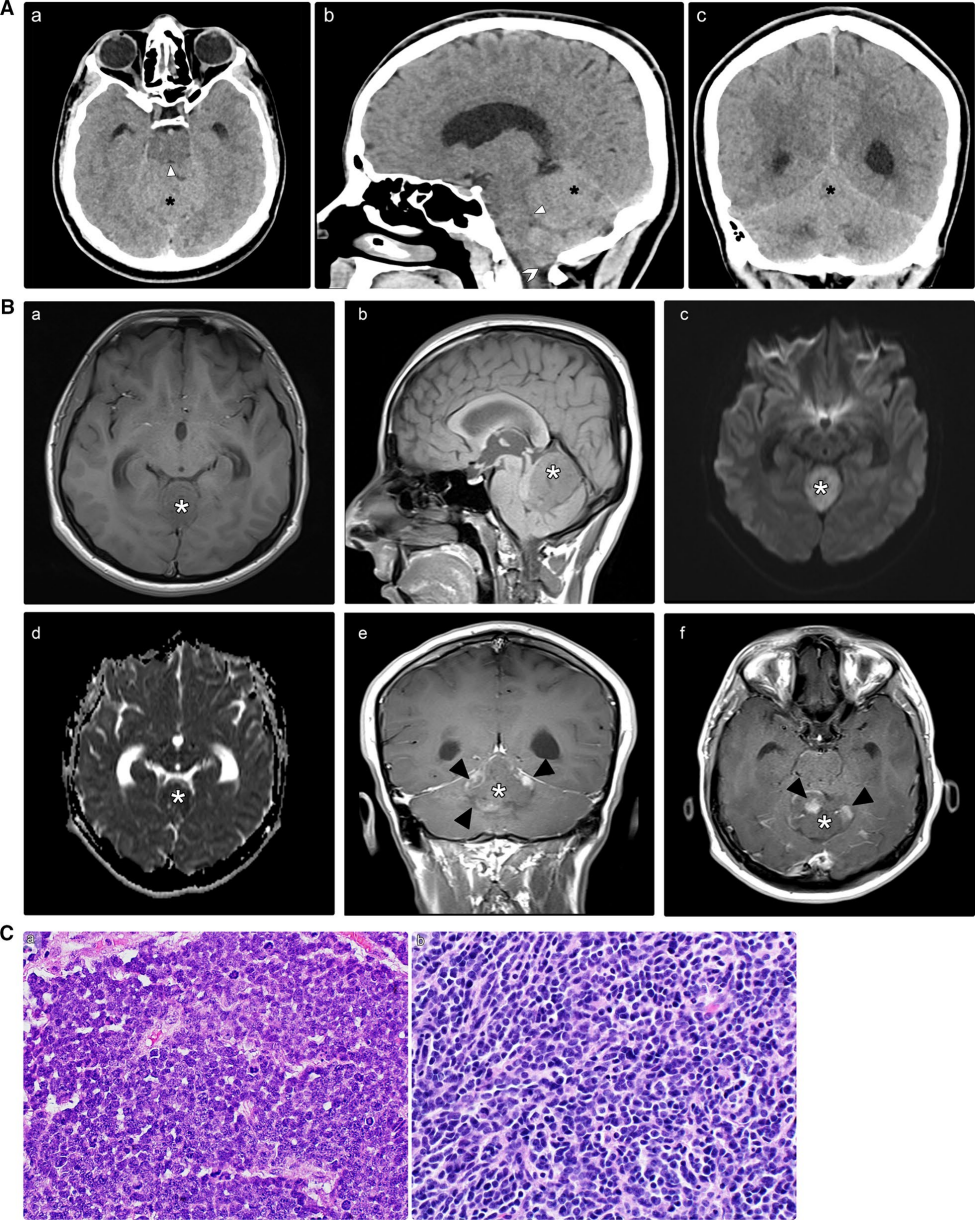
**A** A 17-year-old male was suspected of having an intracranial tumor. Brain CT scan without (a) and with intravenous contrast administration (b, c). There is a left-sided, well-defined, large, hyperdense lateral cerebellar mass (asterisks), with diffuse homogenous enhancement after contrast administration (b, c). The mass induces a mass effect on the cerebellum (black arrowheads), brain stem (benched arrows), and fourth ventricle (white arrowheads). Therefore, blockage causes obstructive hydrocephalus. *CT* computed tomography. **B** Brain MRI without and with intravenous contrast administration. A solid mass lesion involving the tentorial base (asterisks) is depicted. Regarding the gray matter, solid components display an iso intensity on T1-weighted image (a, b), and a high signal intensity on T2-weighted image (not shown). Diffusion-weighted images demonstrate restricted diffusion (c, d). Post-contrast images demonstrate peripheral nodular enhancement (arrowheads) (e, f). Neuroaxis imaging was unremarkable (not shown). *MRI* magnetic resonance imaging. **C** Histopathological analysis following surgery with various magnifying powers. (a) Hematoxylin and eosin staining at different magnifications reveal large neoplastic cells of marked anaplasia and large nuclei with evident nucleoli. (b) The cell wrapping and necrotic phenomena are present. The ultimate diagnosis was medulloblastoma, with a large type of anaplastic cells (WHO grade IV). *WHO* World Health Organization

The tentorial location is considered to be the second most common location of medulloblastomas after the CPA. The presence of medulloblastomas at this location was first described by Becker et al. [22] in 1995. Only four adult cases (8%) were found in the PubMed database in the present search, with an equal male to female ratio (Table 1). Medulloblastomas in this or in the lateral cerebellar location are difficult to differentiate from meningiomas or other pathologies, especially when the medulloblastomas exhibit dural tail signs (Table 2) [10, 23]. Histopathological analysis revealed the SHH-activated subgroup among all cases of medulloblastomas in this location [24]. Overall, a high degree of suspicion should be raised when considering medulloblastomas in the differential diagnoses of an adult patient with an extra-axial tentorial midline mass with atypical features. The prognosis of tentorial medulloblastoma remains uncertain because of the small number of reported cases and the short-term follow-up examination period. We present the case of a 17-year-old male who was diagnosed with the SHH-activated type of medulloblastoma based on imaging and post-surgical pathology results (Fig. 5). One-year follow-up after adjuvant therapy revealed no recurrence.

### Lateral cerebellar location

The lateral cerebellar region, which includes the retro-cerebellar region, is considered the third most common location after the CPA and tentorial regions. This site is bounded beyond the border of the lateral cerebellar hemisphere and also includes the retro-cerebellar site and is marginated by the mastoid part of the temporal and occipital bones. It is an extremely rare location for an extra-axial medulloblastoma. Medulloblastoma cases in this location were first reported by Meshkini et al. [23] in 2014. The reports were found through PubMed during our research and included only three patients (6%, i.e., two adult cases and one pediatric case of medulloblastoma [male:female ratio, 2:1]; Table 1) [24–26].

**Fig. 6:**
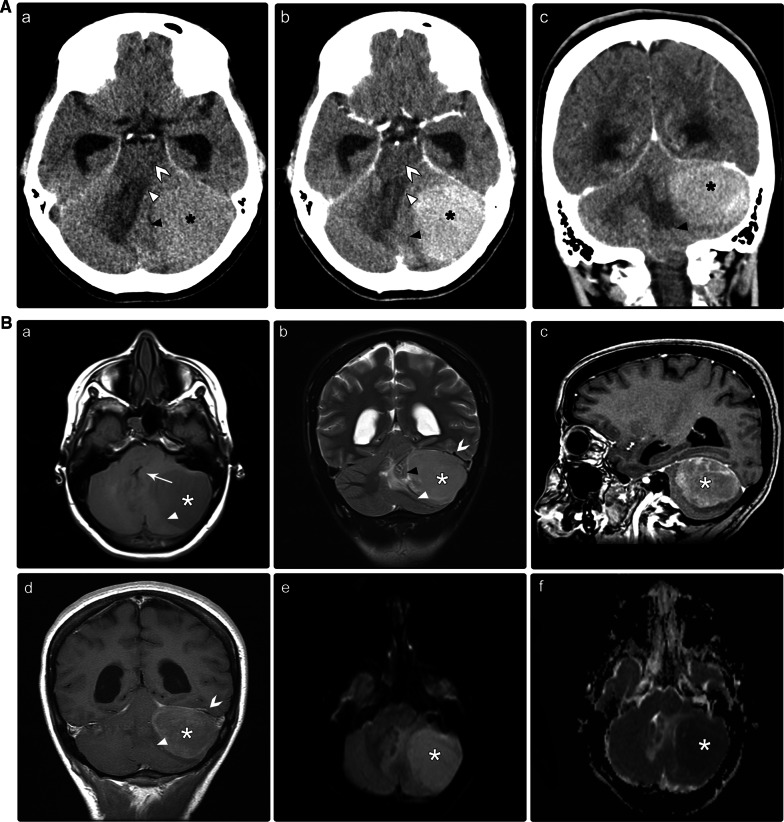

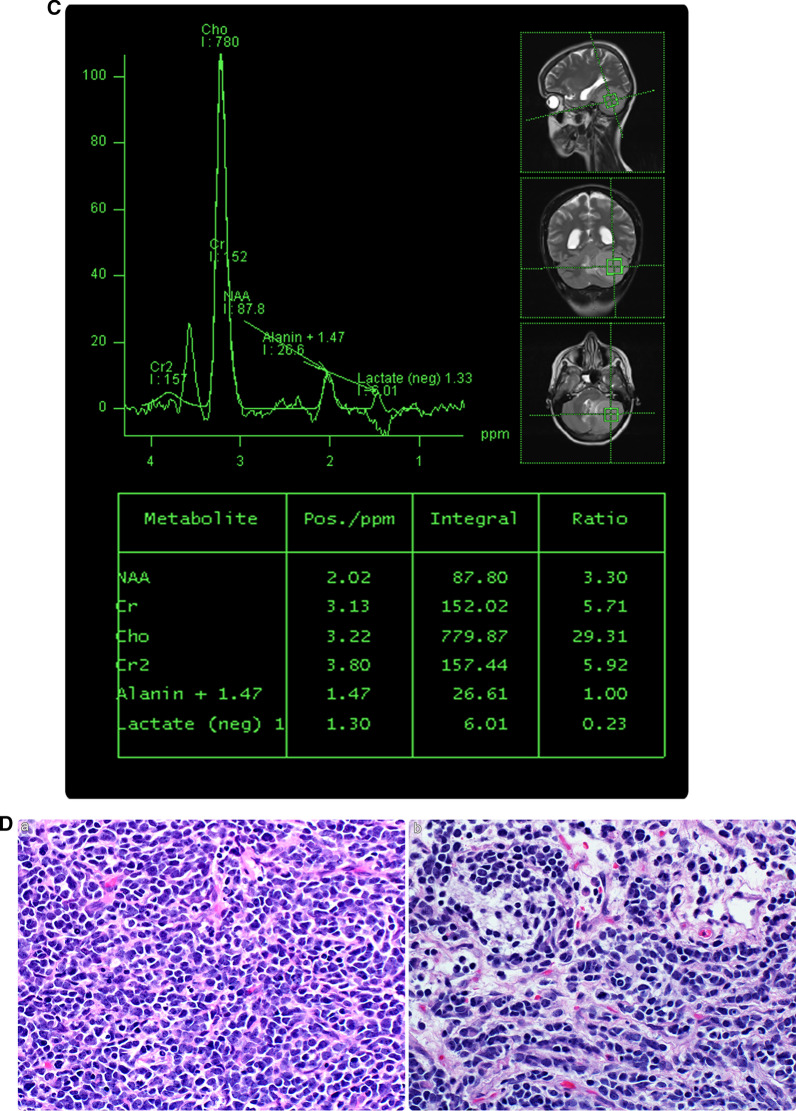
**A** A 17-year-old male suspected of having an intracranial tumor. Brain CT without (a) and with contrast medium (b–d). A left-sided, well-defined, large, hyperdense lateral cerebellar mass (asterisks) with diffuse homogenous enhancement with contrast medium (b–d) exerts mass effect on the fourth ventricle (white arrowheads), cerebellum (black arrowheads), and brain stem (benched arrows). It also causes ballooning of the temporal horns of the lateral ventricles, indicating obstructive hydrocephalus. *CT* computed tomography. **B** Brain MRI without and with intravenous contrast medium. A well-defined mass centered in the lateral cerebellar region (asterisks) is observed, showing hypointense signal on T1-weighted imaging (a) and hyperintense signal on T2-weighted imaging (b). Post-contrast images demonstrate diffuse heterogeneous enhancement (c, d) with restricted diffusion (e, f). The mass causes buckling of the cerebellar parenchyma, folia (white arrowheads). Moreover, a rim of cerebrospinal fluid cleft at the periphery denotes extra-axial localization (black arrowhead; b). It has a mass effect on the brain stem, contralateral cerebellar hemisphere, and fourth ventricle (arrow), resulting in obstructive hydrocephalus. Additionally, it exerts a mass effect over the ipsilateral dural venous sinus system (benched arrows). The neuroaxis imaging finding was unremarkable (not shown). **C** Proton magnetic resonance spectroscopy analysis (single-voxel technique). A prominent choline peak, a high choline/creatinine ratio, and a decreased NAA peak are observed. The taurine peak is not evident at 3.25 or 3.43 ppm. **D** Post-surgery histopathological analysis at various magnifications. (a) Hematoxylin and eosin staining show classic nodular tumor cells. Small basophilic tumor cells exhibit diffuse growth and are arranged in trabeculae, sheets, and nests. (b) The tumor cells have round or oval nuclei with deeply stained chromatin and a high nuclear-cytoplasmic ratio with mitotic figures and partial necrosis. The final diagnosis is medulloblastoma of a nodular desmoplastic type (WHO grade IV). *WHO* World Health Organization

Medulloblastomas in this location are assumed to arise from the remnants of the external granular layer in the cerebellar hemisphere. Similar to medulloblastomas at the tentorial location, it is difficult to differentiate medulloblastomas in this region from meningiomas or other pathologies, especially when medulloblastomas exhibit dural tail signs, which is usually observed in meningiomas in this or in the tentorial location (Table 2). The lateral cerebellar medulloblastoma prognosis remains uncertain because of the small number and short-term follow-up period of the reported cases [10, 24]. We present the case of a 17-year-old male diagnosed with medulloblastoma of a nodular desmoplastic type based on imaging and post-surgical pathology results (Fig. 6). Three-year follow-up after adjuvant therapy revealed no recurrence.

### Foramen magnum location

**Fig. 7:**
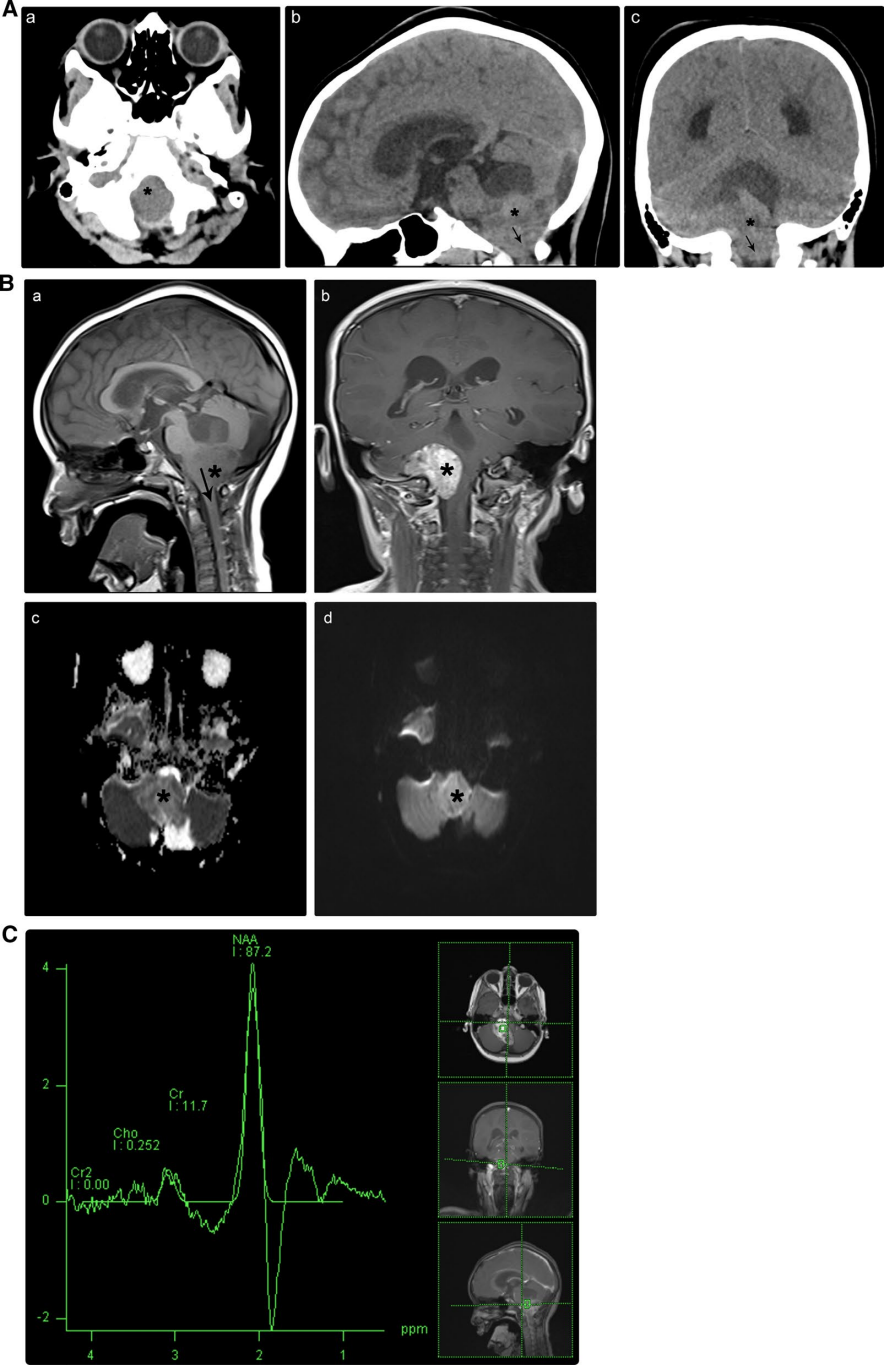
**A** A 15-year-old girl presented with nausea and vomiting. Brain CT scan without contrast administration in axial (a), sagittal (b), and coronal (c) reformats. A well-defined heterogeneous mass was observed centered in the foramen magnum with a marginal extension on the right side (asterisks). It shows homogenous density and extends below the foramen magnum caudally (arrows). It induces a mass effect on the inferior aspect of the cerebellar hemisphere and medulla oblongata, resulting in obstructive hydrocephalus. *CT* computed tomography. **B** Brain MRI without and with intravenous contrast medium. The figure indicates a lobulated, heterogeneous, solid mass, centered within the foramen magnum (asterisks). This lesion shows mixed heterogeneous intensity with predominantly iso-to-hypointensity on T1-weighted imaging (a), high signal intensity on T2-weighted imaging (not shown), and heterogeneous enhancement after contrast medium administration (b). Diffusion-weighted images demonstrate restricted diffusion (c, d). It induces a mass effect over the medulla oblongata with resultant hydrocephalus (arrow; a). The findings of neuroaxis imaging were unremarkable (not shown). *MRI* magnetic resonance imaging. **C** Proton magnetic resonance spectroscopy analysis (single-voxel technique) for a female pediatric patient presented with nausea and vomiting. There is a high NAA peak, a low choline peak, and choline to creatinine ratio. The taurine peak is not evident at 3.25 or 3.43 ppm. *NAA* N-acetyl aspartate

The foramen magnum is the largest foramen of the skull, which is located in the most inferior part of the cranial pit as a part of the occipital bone [27]. The foramen magnum location has not been reported previously as a separate origin of extra-axial medulloblastomas. Nevertheless, many of the previously reviewed locations exhibit medulloblastoma extension through the foramen magnum without a clear sign of origin [28]. This is particularly true for lesions that originate from the CPA with caudal descent [28, 29]. These lesions might present as a physical pit in the brain (i.e., a morphological change), and the diagnostic neuroradiologist should be attentive for such an exceedingly uncommon occurrence [30]. A multidisciplinary approach that considers the clinic-radio-pathological correlations could lead to a more accurate diagnosis. We present the case of a 15-year-old female with a nonspecific controversial diagnosis of low-grade tumor who died before operating on her (Fig. 7).

## Advanced medical imaging for intra-axial and extra-axial medulloblastoma

Recently, there have been tremendous advances in diagnostic neuro-oncology. Accordingly, it is currently possible to detect this devastating tumor at an earlier stage. For example, in vivo detection of the receptor status using positron emission tomography (PET) scan on mothers has enabled diagnosticians to anticipate the potential consequences.

### Updates in radionuclide imaging

- Although at present, radionuclide imaging is not the primary diagnostic modality for intracranial tumors, primarily limited by its low specificity and low spatial resolution. In 1998, Müller et al. [31] evaluated potential applications of radionuclide-based imaging techniques for children with medulloblastoma. Radionuclide imaging is useful in detecting various intracranial tumors, including meningioma, pituitary adenoma, hemangioblastomas, gliomas, and medulloblastomas [32, 33]. Furthermore, the continued growth of tumor-specific radiotracers makes it very functional. For instance, a new radiotracer, 3-deoxy-3-[18F] fluorothymidine (FLT), is a molecule that is preceded by thymidine kinase-1 during phase S of mitosis. This tracer is distinct in that there is an uptake in setting a disrupted blood–brain barrier, which makes it very helpful and specific in detecting and determining the grade of brain tumors since higher-grade cancers are associated with greater disruption of the blood–brain barrier [34, 35].
- Radionuclide imaging has a limited potential in identifying medulloblastoma from differentiated diagnoses. However, the fusion of CT or MRI images with PET images can improve PET’s ability to diagnose and distinguish post-radiotherapy changes from tumor recurrence [36]. This is employed by the increase in cellular activity and glucose uptake in neoplasms relative to normal cells [37]. For instance, medulloblastomas have a limited potential for uptake in thallium-201 single-photon emission computed tomography and fluorodeoxyglucose PET (tumor-to-normal uptake ratio) [36, 38].
- Radionuclide imaging shows a limited potential capability in diagnoses and identification of medulloblastomas compared with differential diagnoses, and medulloblastomas have a limited potential for uptake in thallium-201 single-photon emission CT and fluorodeoxyglucose PET (tumor-to-normal uptake ratio) [39].

### Updates in advanced MR imaging

**Fig. 8:**
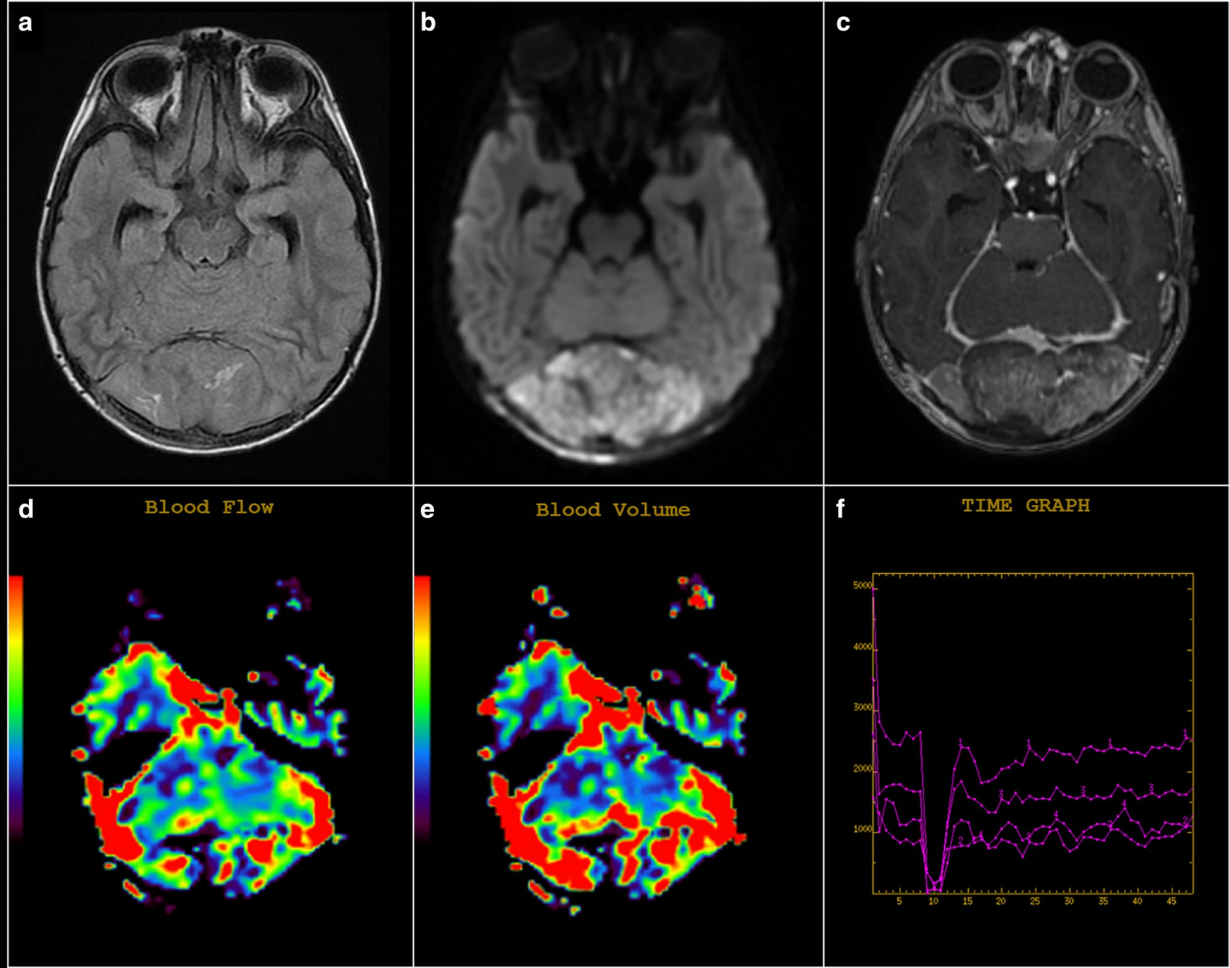
Advanced MRI imaging for medulloblastoma. Axial FLAIR (**a**) and DWI (**b**) display an extra-axial mass occupying the lateral and retrocerebellar regions, demonstrating a high signal in FLAIR with restricted diffusion in DWI. Axial T1 after contrast administration (**c**) demonstrates strong heterogeneous contrast enhancement of the mass. DSC-MR perfusion maps (**d**–**f**) show increased vascularization in the mass, with increased blood flow (**d**) and high value of rCBV (**e**) of the mass than the normal parenchyma. *DWI* diffusion-weighted image, *DSC* dynamic susceptibility contrast-enhanced, *rCBV* relative cerebral blood volume

- Dynamic perfusion parameters have recently been evaluated for their potential diagnostic roles in neuro-oncology, especially for lesions of predominantly nonnecrotic solid tumors [27, 40, 41].
- Dynamic susceptibility contrast imaging of medulloblastomas has revealed increased permeability, with the cerebral blood volume ratios close to 1 [40], particularly in desmoplastic cases. The findings significantly contradict those obtained from other differential diagnoses of enhancing the posterior fossa tumors [40].
- Medulloblastomas are considered as tumors with the greatest relative tumor blood flow (Fig. 8), which can be used to distinguish medulloblastomas from pilocytic astrocytomas. The observation may complement diffusion-weighted imaging and help accurately distinguish these tumors [27].
- Finally, it is noteworthy that the characteristic arterial spin-labeling perfusion patterns have been studied among diverse pathologic types of brain tumors in children. These studies have revealed that the maximum relative tumor blood flow of high-grade tumors (grades III and IV) is significantly higher than that of low-grade tumors (grades I and II) [27, 41].
- Quantitative Apparent Diffusion Coefficient (ADC) value analysis can facilitate preoperative identification of medulloblastoma from its differentials, as well as grading of pediatric medulloblastoma [42, 43]. Further, it can facilitate optimal surgical treatment planning, with reduction of surgery-induced morbidity [43, 44].
- The ADC ratio—the proportion between the mean ADC observed in the tumor and the mean ADC observed in the contralateral white matter—is a simple tool used to distinguish juvenile pilocytic astrocytomas, ependymomas, and medulloblastomas [45]. In particular, the ADC ratio cut-off value was set below 1, as 1 was characteristic for medulloblastoma with 100% sensitivity and 90% specificity [44, 46].
- MRS based on the metabolic pattern can be used to identify medulloblastomas (Figs. 6C, 7C). A recent observational study of 111 medulloblastoma patients revealed a predictive accuracy of 95% for the SHH-activated group, 78% for group 4, 56% for group 3, and 41% for the WNT-activated group. Reflecting on specific preoperative features of MRI/MRS enabled the prediction of a molecular subgroup of medulloblastoma using a five-metabolite subgroup classifier (creatine, myoinositol, taurine, aspartate, and lipid) [46].
- MR perfusion is a distinguishing modality for posterior fossa diagnosis. In 2014, Yeom et al. [39] clarified the effect of maximal relative tumor blood flow (rTBF) on tumor grade (low vs. high grade) and found a difference in the range of rTBF between medulloblastomas (0.98 and 4.97) and pilocytic tumors (1.05 ± 0.18). In addition, medulloblastomas showed higher rTBF values compared to ependymomas, with an overlap between these two tumors because of the perfusion variability of the former [39]. Koob et al. [47] quantified the perfusion map parameters, i.e., the tumor-to-parenchyma ratios for relative enhancement, maximum enhancement, maximum relative enhancement, time to peak, and AUC values for medulloblastoma, which was significantly higher than ependymoma parameters (p < 0.05). A maximum cut-off enhancement value of 100.25 was used to distinguish between medulloblastoma and ependymoma (sensitivity 90.9%, specificity 100%) [47]. In 2020, Gaudino et al. [48] examined the data of 246 brain tumor patients by calculating the relative cerebral blood volume (rCBV) and the mean percentage of signal recovery (PSR). The optimum rCBV value threshold was 1.77 (sensitivity, 100%; specificity, 85%; PPV, 84%; NPV, 100%) [48].

## Conclusions

Clinical assessment and neuroimaging findings are insufficient in obtaining an accurate preoperative diagnosis of extra-axial medulloblastomas because of shared features between medulloblastomas and other common pathologies. Most reported cases of posterior fossa extra-axial medulloblastomas are located in the CPA. Radionuclide and arterial spin-labeling imaging are newer and significantly useful techniques for the diagnosis and recurrence detection of medulloblastomas. Moreover, the somatostatin receptor subtype 2 might be a potential prognostic marker and therapeutic target for medulloblastomas. We believe that cases of medulloblastomas are likely under-reported because of publication bias and a tendency to report usual tumors at unusual locations. The information provided in this review would help establish a more accurate understanding of these lesions as one of the essential roles of a neuroradiologist is to detect the tumor and possible subarachnoid spread, which determines and guides the surgical approach.

## Data Availability

Not applicable. This is a review of publicly available information.

## Abbreviations

CPA: Cerebellopontine angle
WNT: Wingless-activated
SHH: Sonic Hedgehog-activated
CT: Computed tomography
MRI: Magnetic resonance imaging
ADC: Apparent diffusion coefficient

## References

[1] Menon G, Krishnakumar K, Nair S (2008). Adult medulloblastoma: clinical profile and treatment results of 18 patients. J Clin Neurosci.

[2] Kumar R, Achari G, Banerjee D, Chhabra D (2001). Uncommon presentation of medulloblastoma. Childs Nerv Syst.

[3] Louis DN, Perry A, Reifenberger G (2016). The 2016 World Health Organization classification of tumors of the central nervous system: a summary. Acta Neuropathol.

[4] Stevenson L, Echlin F (1934). Nature and origin of some tumors of the cerebellum: medulloblastoma. Arch NeurPsych.

[5] Spina A, Boari N, Gagliardi F, Franzin A, Terreni MR, Mortini P (2013). Review of cerebellopontine angle medulloblastoma. Br J Neurosurg.

[6] Wilke M, Eidenschink A, Müller-Weihrich S, Auer DP (2001). MR diffusion imaging and 1H spectroscopy in a child with medulloblastoma: a case report. Acta Radiol.

[7] Brandão LA, Poussaint TY (2017). Posterior fossa tumors. Neuroimaging Clin N Am.

[8] Blüml S, Margol AS, Sposto R (2015). Molecular subgroups of medulloblastoma identification using noninvasive magnetic resonance spectroscopy. Neuro Oncol.

[9] Fallah A, Banglawala SM, Provias J, Jha NK (2009). Extra-axial medulloblastoma in the cerebellopontine angle. Can J Surg.

[10] Furtado SV, Venkatesh PK, Dadlani R, Reddy K, Hegde AS (2009). Adult medulloblastoma and the “dural-tail” sign: rare mimic of a posterior petrous meningioma. Clin Neurol Neurosurg.

[11] Pomeroy SL, Tamayo P, Gaasenbeek M (2002). Prediction of central nervous system embryonal tumor outcome based on gene expression. Nature.

[12] Bhaskar MK, Jaiswal M, Ojha BK (2017). Extra-axial cerebellopontine angle medulloblastoma in an infant. Pediatr Neurosurg.

[13] Sure U, Berghorn WJ, Bertalanffy H (1995). Staging, scoring and grading of medulloblastoma. a postoperative prognosis predicting system based on the cases of a single institute. Acta Neurochir (Wien).

[14] Ammirati M, Scerrati A, Signorelli F (2016). Surgical techniques in benign extra-axial tumors. From bench to bedside. Trauma, tumors, spine, functional neurosurgery.

[15] Stricsek GP, Evans JJ, Farrell CJ, Kumar M, Levine J, Schuster J, Kofke A (2018). Cerebellopontine angle tumors. Neurocritical care management of the neurosurgical patient.

[16] Yamada S, Aiba T, Hara M (1993). Cerebellopontine angle medulloblastoma: case report and literature review. Br J Neurosurg.

[17] Akay KM, Erdogan E, Izci Y, Kaya A, Timurkaynak E (2003). Medulloblastoma of the cerebellopontine angle. Neurol Med Chir.

[18] Jaiswal AK, Mahapatra AK, Sharma MC (2004). Cerebellopointine angle medulloblastoma. J Clin Neurosci.

[19] Xia H, Zhong D, Wu X, Li J, Yang Y, Sun X (2019). Medulloblastomas in cerebellopontine angle: epidemiology, clinical manifestations, imaging features, molecular analysis and surgical outcome. J Clin Neurosci.

[20] Wu T, Qu PR, Zhang S (2020). The clinical treatment and outcome of cerebellopontine angle medulloblastoma: a retrospective study of 15 cases. Sci Rep.

[21] Adeeb N, Mortazavi MM, Tubbs RS, Cohen-Gadol AA (2012). The cranial dura mater: a review of its history, embryology, and anatomy. Childs Nerv Syst.

[22] Becker RL, Becker AD, Sobel DF (1995). Adult medulloblastoma: review of 13 cases with emphasis on MRI. Neuroradiol.

[23] Meshkini A, Vahedi A, Meshkini M, Alikhah H, Naghavi-Behzad M (2014). Atypical medulloblastoma: a case series. Asian J Neurosurg.

[24] Doan NB, Patel M, Nguyen HS (2018). A rare extra-axial midline tentorial adult medulloblastoma with dural-tail sign mimicking a meningioma. Asian J Neurosurg.

[25] Presutto E, Chappell M, Fullmer J, Ezhapilli S (2018). Posterior fossa medulloblastoma in an atypical extra-axial location: a case report. Radiol Case Rep.

[26] Chung EJ, Jeun SS (2014). Extra-axial medulloblastoma in the cerebellar hemisphere. J Korean Neurosurg Soc.

[27] Dallery F, Bouzerar R, Michel D (2017). Perfusion magnetic resonance imaging in pediatric brain tumors. Neuroradiol.

[28] Faleiro RM, de Souza Moraes VV, de Seixas Alves MT (2016). Extra-axial cerebello-pontine angle medulloblastoma. Arq Bras Neurocir.

[29] Goudihalli SR, Pathak A, Brar R, Mundi I (2018). Reappraisal of cerebellopontine angle medulloblastomas: report of a fatal case and lessons learned. Interdiscip Neurosurg.

[30] Zoltán L (1974). Die tumoren im foramen occipitale magnum. Acta Neurochir.

[31] Müller HL, Fruhwald MC, Scheubeck M (1998). A possible role for somatostatin receptor scintigraphy in the diagnosis and follow-up of children with medulloblastoma. J Neurooncol.

[32] Sharma P, Mukherjee A, Bal C, Malhotra A, Kumar R (2013). Somatostatin receptor-based PET/CT of intracranial tumors: a potential area of application for 68Ga-DOTA peptides?. Am J Roentgenol.

[33] Guyotat J, Champier J, Pierre GS (2001). Differential expression of somatostatin receptors in medulloblastoma. J Neurooncol.

[34] Chen W, Cloughesy T, Kamdar N (2005). (2005) Imaging proliferation in brain tumors with 18F-FLT PET: comparison with 18F-FDG. J Nucl Med.

[35] Muzi M, Spence AM, O'Sullivan F (2006). Kinetic analysis of 3′-deoxy-3′-18F-fluorothymidine in patients with gliomas. J Nucl Med.

[36] Horky LL, Treves ST (2011). PET and SPECT in brain tumors and epilepsy. Neurosurg Clin N Am.

[37] Dhermain FG, Hau P, Lanfermann H, Jacobs AH, van den Bent MJ (2010). Advanced MRI and PET imaging for assessment of tratment response in patients with gliomas. Lancet Neurol.

[38] Remke M, Hering E, Gerber NU (2013). Somatostatin receptor subtype 2 (sst 2) is a potential prognostic marker and a therapeutic target in medulloblastoma. Child Nerv Syst.

[39] Yeom KW, Mitchell LA, Lober RM (2014). Arterial spin-labeled perfusion of pediatric brain tumors. Am J Neuroradiol.

[40] Yamasaki F, Kurisu K, Satoh K (2005). Apparent diffusion coefficient of human brain tumors at MR imaging. Radiology.

[41] Reddy N, Ellison DW, Soares BP, Carson KA, Huisman TA, Patay Z (2020). Pediatric posterior fossa medulloblastoma: the role of diffusion imaging in identifying molecular groups. J Neuroimaging.

[42] Al-Sharydah AM, Al-Arfaj HK, Al-Muhaish HS (2019). Can apparent diffusion coefficient values help distinguish between different types of pediatric brain tumors?. Eur J Radiol Open.

[43] Esa MM, Mashaly EM, El-Sawaf YF, Dawoud MM (2020). Diagnostic accuracy of apparent diffusion coefficient ratio in distinguishing common pediatric CNS posterior fossa tumors. Egypt J Radiol Nucl Med.

[44] Dasgupta A, Gupta T, Pungavkar S (2019). Nomograms based on pre-operative multiparametric magnetic resonance imaging for prediction of molecular subgrouping in medulloblastoma: results from a radiogenomics study of 111 patients. Neuro Oncol.

[45] Domínguez-Pinilla N, de Aragón AM, Tapias SD (2016). Evaluating the apparent diffusion coefficient in MRI studies as a means of determining paediatric brain tumour stages. Neurología (English Edition).

[46] Pant I, Chaturvedi S, Gautam VK, Pandey P, Kumari R (2016). Extra-axial medulloblastoma in the cerebellopontine angle: report of a rare entity with review of literature. J Pediatr Neurosci.

[47] Koob M, Girard N, Ghattas B (2016). The diagnostic accuracy of multiparametric MRI to determine pediatric brain tumor grades and types. J Neurooncol.

[48] Gaudino S, Benenati M, Martucci M (2020). Investigating dynamic susceptibility contrast-enhanced perfusion-weighted magnetic resonance imaging in posterior fossa tumors: differences and similarities with supratentorial tumors. La Radiol Med.

[49] Gil-Salu JL, Rodriguez-Pena F, Lopez-Escobar M, Palomo MJ (2004). Medulloblastoma presenting as an extra-axial tumor in the cerebellopontine angle. Neurocirugia.

[50] Singh M, Cugati G, Symss NP, Pande A, Vasudevan MC, Ramamurthi R (2011). Extra axial adult cerebellopontine angle medulloblastoma: an extremely rare site of tumor with metastasis. Surg Neurol Int.

[51] Bahrami E, Bakhti S, Fereshtehnejad SM, Parvaresh M, Khani MR (2014). Extra-axial medulloblastoma in cerebello-pontine angle: a report of a rare case with literature review. Med J Islam Repub Iran.

[52] Mehta JS, Sharr MM (1998). An unusual cause of acute labyrinthine failure. J Laryngol Otol.

[53] Ahn MS, Jackler RK (1997). Exophytic brain tumors mimicking primary lesions of the cerebellopontine angle. Laryngoscope.

[54] Naimur-Rahman JA, Al-Rayess M, Jamjoom ZA (2000). Cerebellopontine angle medulloblastoma. Br J Neurosurg.

[55] Izycka-Swieszewska E, Debiec-Rychter M, Kloc W (2003). Primitive neuroectodermal tumor in the cerebellopontine angle with isochromosome 17q presenting as meningioma in a woman 26 years of age. Clin Neuropathol.

[56] Park SY, Kim JH, Kim KT (2004). A case of medullomyoblastoma of cerebellopontine angle mimicking acoustic neuroma. Yonsei Med J.

[57] Santagata S, Kesari S, Black PM, Chan JA (2007). Anaplastic variant of medulloblastoma mimicking a vestibular schwannoma. J Neurooncol.

[58] Nyanaveelan M, Azmi A, Saffari M, Banu SK, Suryati MY, Jeyaledchumy M (2007). Cerebellopontine angle medulloblastoma. Med J Malays.

[59] Yoshimura J, Nishiyama K, Fukuda M, Watanabe M, Igarashi H, Fujii Y (2009). Adult cerebellopontine angle medulloblastoma originating in the pons mimicking focal brainstem tumor. J Neuroimaging.

[60] Cugati G, Singh M, Symss N, Pande A, Chakravarthy V, Ramamurthi R (2011). Extra-axial cerebello pontine angle medulloblastoma: a rare site of tumor. Indian J Med Paediatr Oncol.

[61] Kumar R, Bhowmick U, Kalra SK, Mahapatra AK (2008). Pediatric cerebellopontine angle medulloblastomas. J Pediatr Neurosci.

